# Supplementary material for: Storm surge and ponding explain mangrove dieback in southwest Florida following Hurricane Irma
Source: Nat Commun. 2021 Jun 28;12:4003. doi: 10.1038/s41467-021-24253-y (PMC8238932; doi:10.1038/s41467-021-24253-y)
Supplement: Supplementary file 1 — Supplementary Information [file 41467_2021_24253_MOESM1_ESM.pdf]

Supplementary Information for “Storm surge and ponding explain mangrove dieback in southwest Florida following Hurricane Irma” by Lagomasino et al.

Supplementary Figures

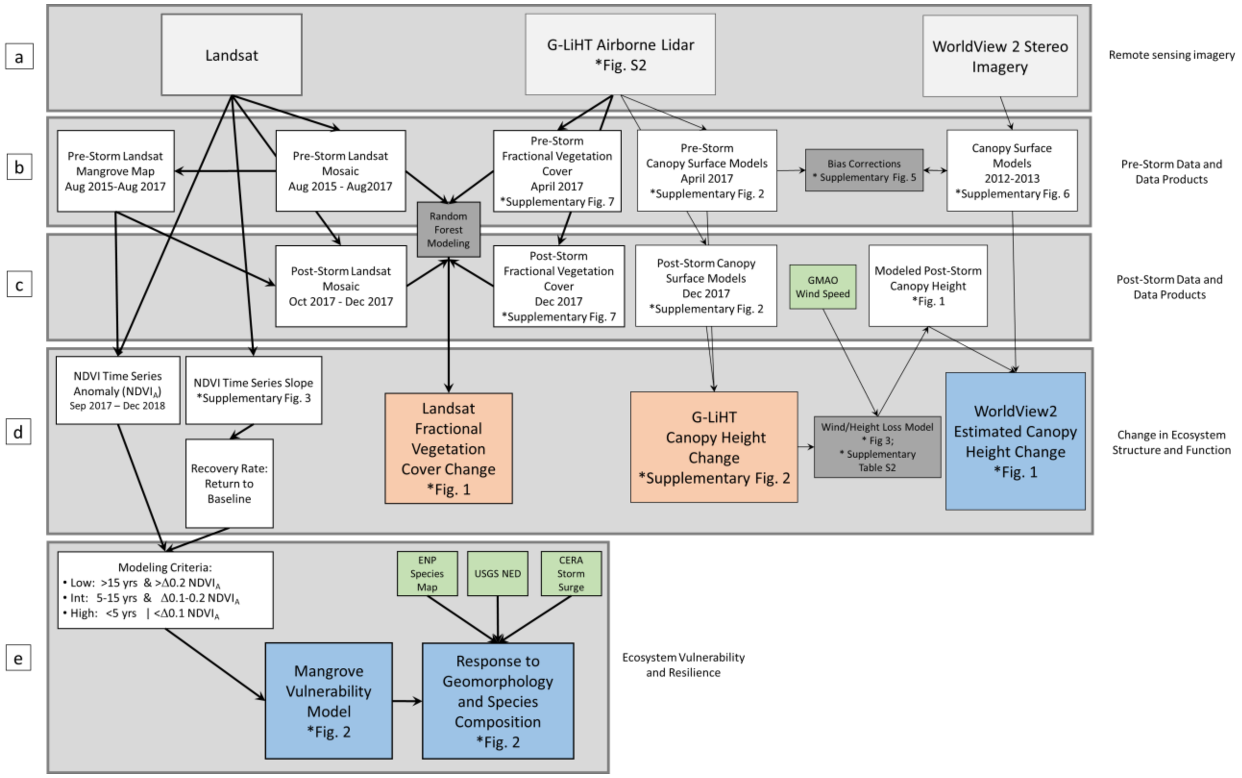

Supplementary Figure 1. (a) This study combined airborne and satellite remote sensing data to quantify changes in mangrove forest structure and function from Hurricane Irma across southwest Florida. (b) Pre-storm forest conditions were generated using a combination of Landsat imagery, NASA G-LiHT lidar collected in April 2017, and high-resolution satellite stereo imagery. (c) Storm impacts were then estimated using a combination of cyclone wind speed, post-storm G-LiHT lidar, and post-storm Landsat data. (d) The changes to the ecosystem following the storm were then calculated from the difference between pre- and post-storm observations. (e) Lastly, the change and recovery data were used to develop a Mangrove Vulnerability model where physical and ecological attributes were then characterized. Green boxes represent supplemental dataset not directly derived by this study. Dark grey shaded boxes represent models that combine G-LiHT lidar with satellite or other spatial datasets. White boxes represent intermediate processing steps for dataset generation. Orange boxes indicate disturbance maps. Blue boxes represent the primary data outputs and disturbance maps derived in this study. Asterisks denote the use of derived data products in main text and supplemental figures.

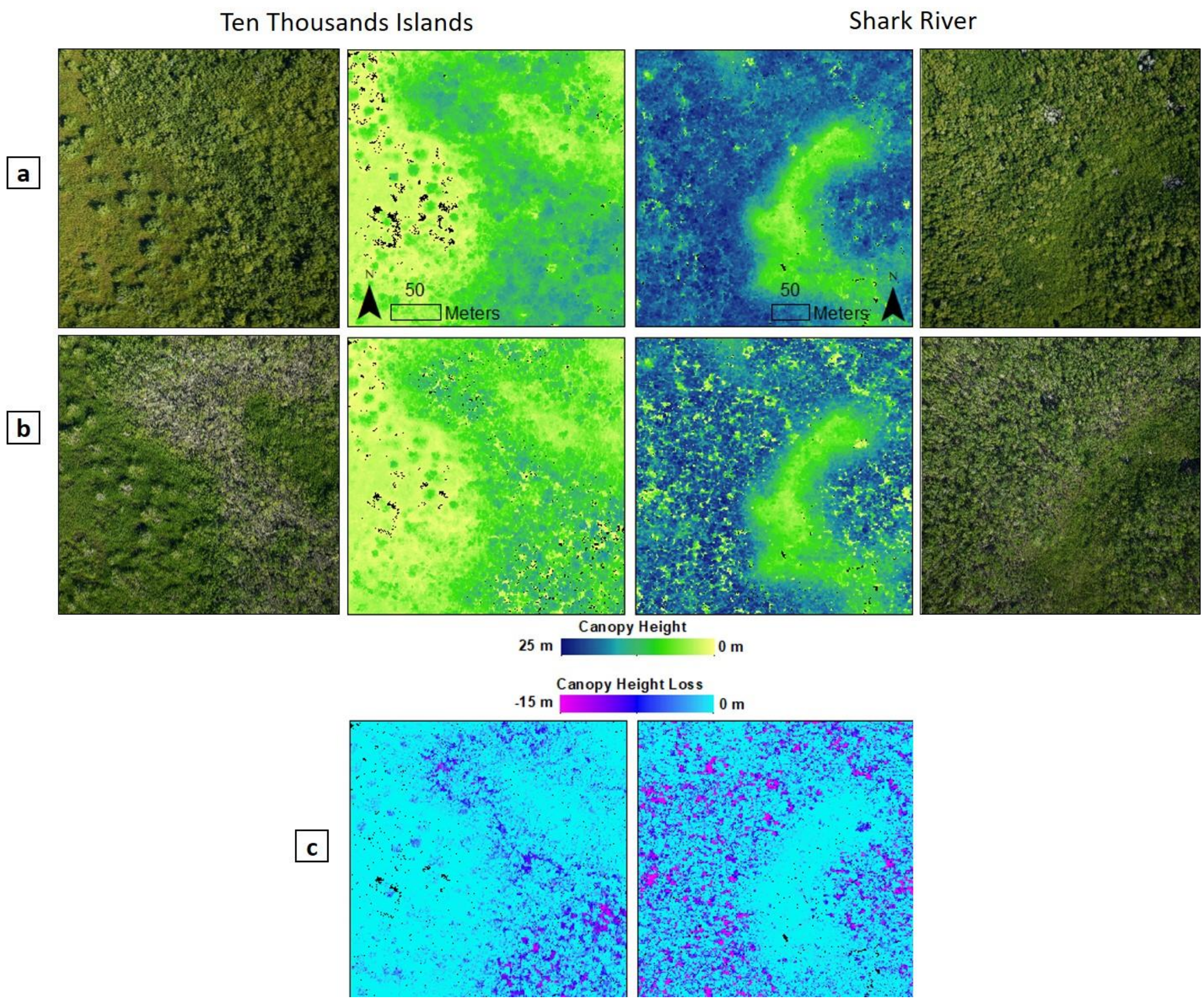

Supplementary Figure 2. Repeat airborne lidar data captured fine-scale patterns of mangrove forest damage from Hurricane Irma, with greater height losses in taller forests. (a) Pre-storm lidar-derived canopy height models for a short and tall mangrove forests in Ten Thousand Islands and Shark River Estuary, respectively. High-resolution photos are provided for reference. (b) Post-storm lidar-derived canopy height models. (c) Estimated canopy height loss in both forest statures.

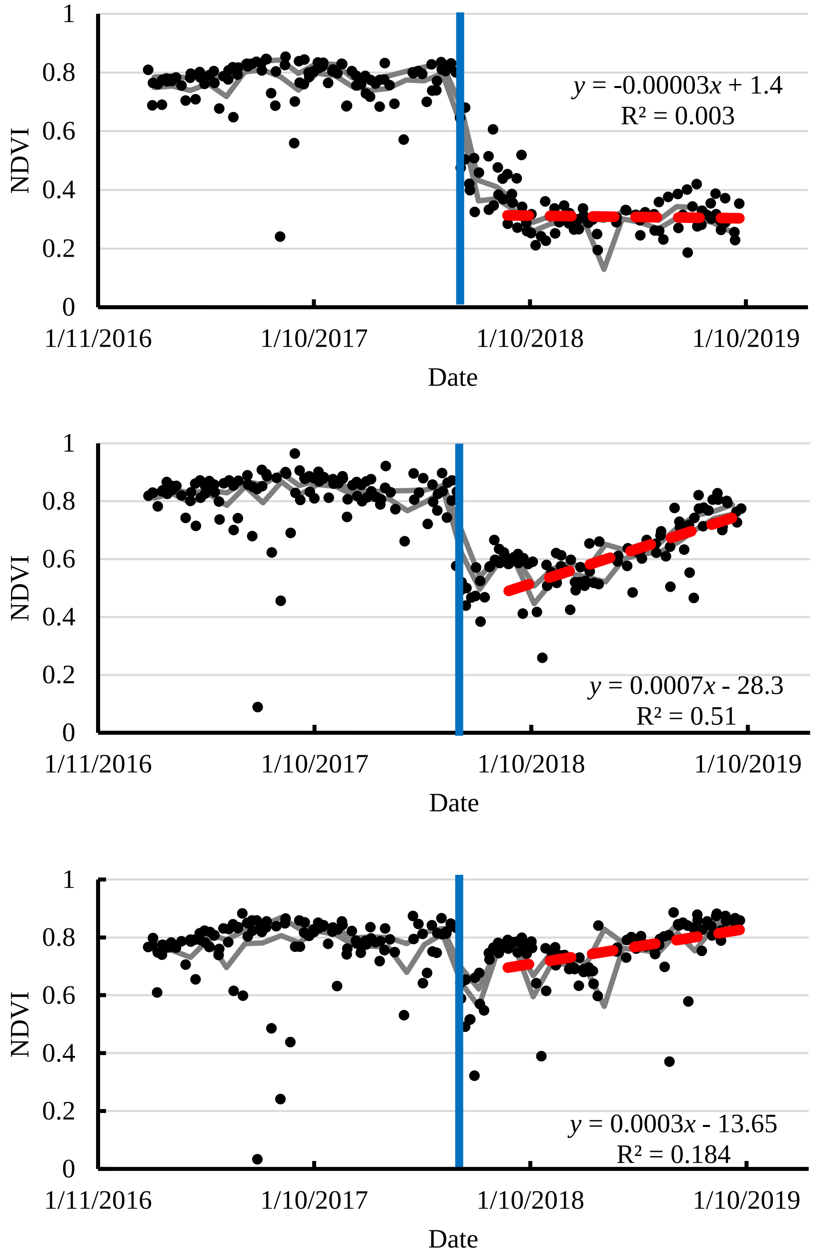

Supplementary Figure 3. Normalized Difference Vegetation Index (NDVI) time series for mangrove forests categorized as (a) low resilience, (b) intermediate resilience, (c) and high resilience. Low resilience was marked by a significant and persistent drop in NDVI after the storm with little to no indication of recovery. Conversely, the intermediate and high resilience exhibited disturbance with signs of recovery in the 15-month period after Hurricane Irma. The black dots represent the average of 50 randomly sampled points within each resilience class. The grey lines denote the monthly 95% confidence interval boundaries. The blue solid lines mark the date Hurricane Irma struck South Florida, September 10, 2017. Red dashed lines represent the NDVI slope used to estimate recovery trajectory using 2018 imagery.

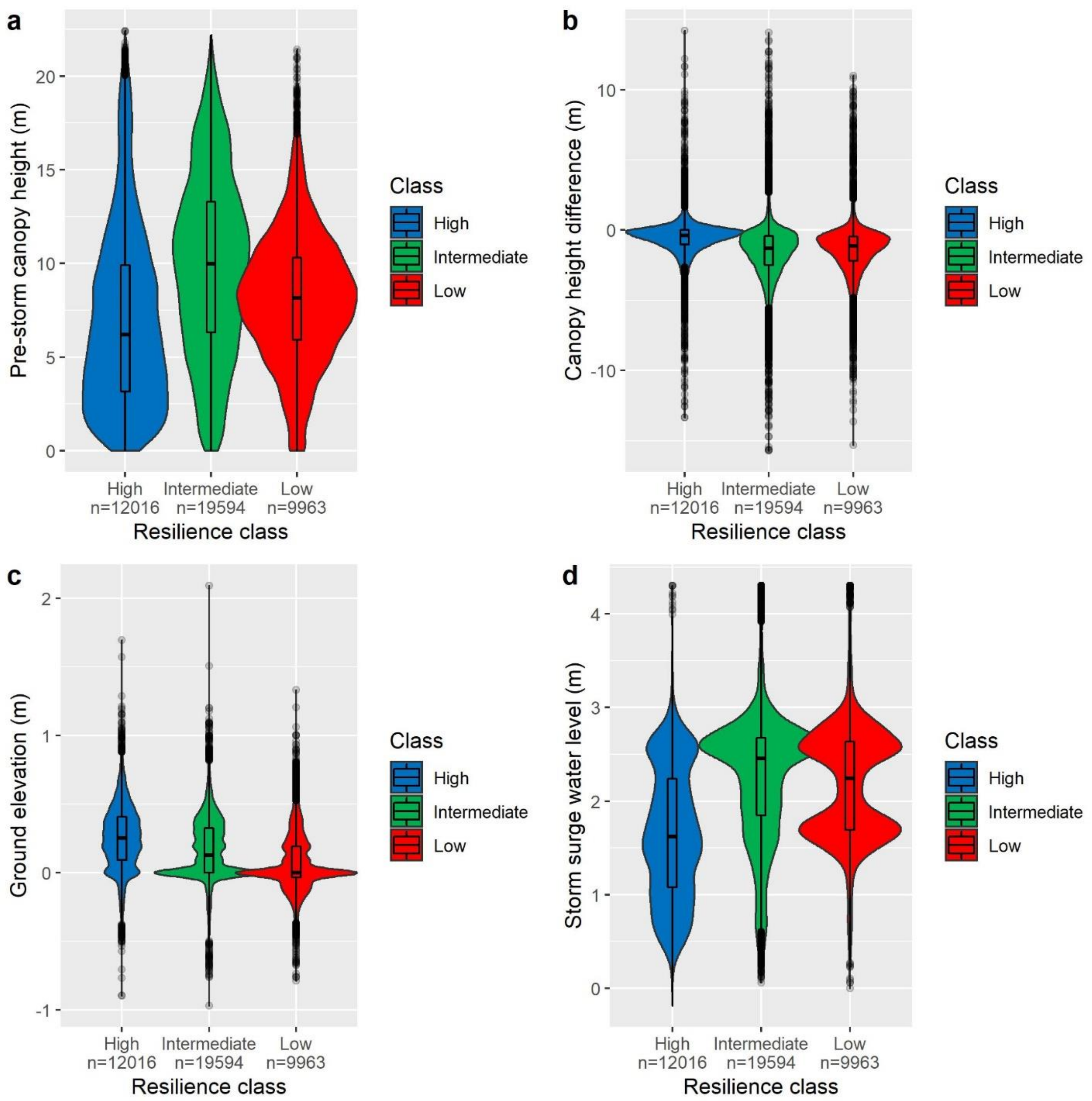

Supplementary Figure 4. The environmental setting of mangroves that were characterized as low resilience were significantly different than the areas that exhibited recovery. Random stratified sampling points were used to extract spatially continues data represent canopy structure and elevation. (a) Pre-storm canopy height measured by G-LiHT in April 2017. (b) Canopy height loss measured by G-LiHT in December 2017. (c) Ground elevation from the National Elevation Dataset. (d) Coastal Emergency Risks Assessment (CERA) storm surge water level over ground. Box plots represent the median, first quartile, third quartile, and 1.5 times the interquartile range, and outliers.

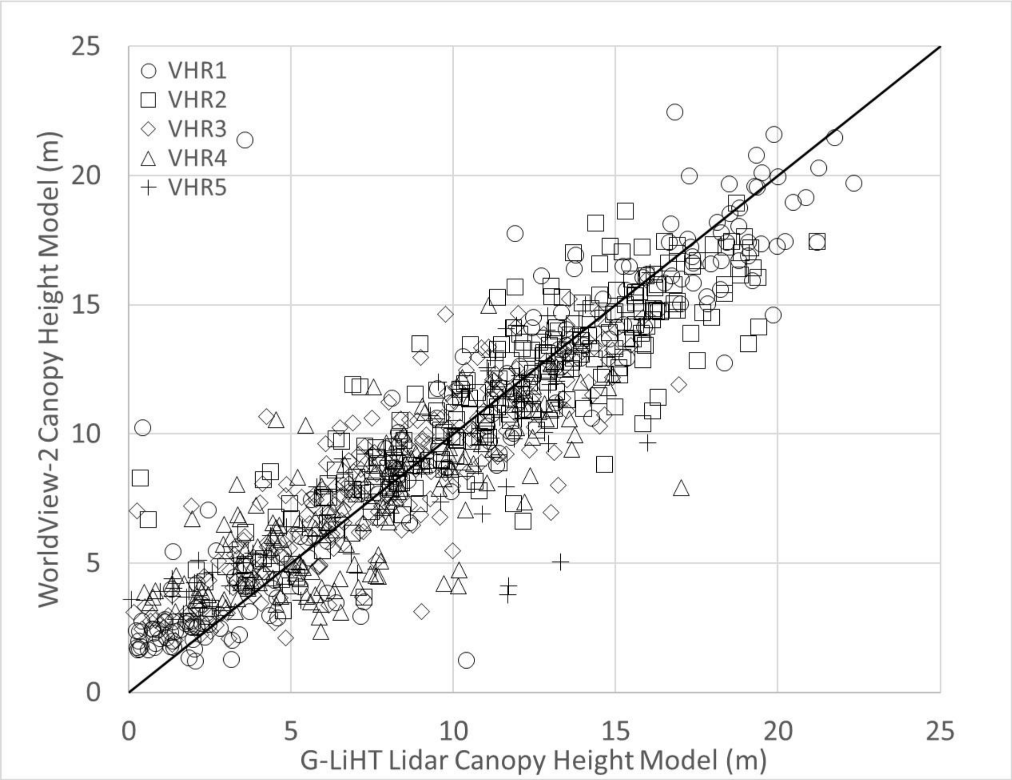

Supplementary Figure 5. G-LIHT lidar data were used to correct canopy surface models from very high-resolution (VHR) WorldView 2 stereo satellite imagery to estimate mangrove canopy heights across the study domain. Each VHR image was independently bias-corrected, and the total estimated root mean square error (RMSE) between lidar and WorldView canopy height estimates was 2.2 m. The extent of each VHR image swath is shown in Supplementary Figure 6.

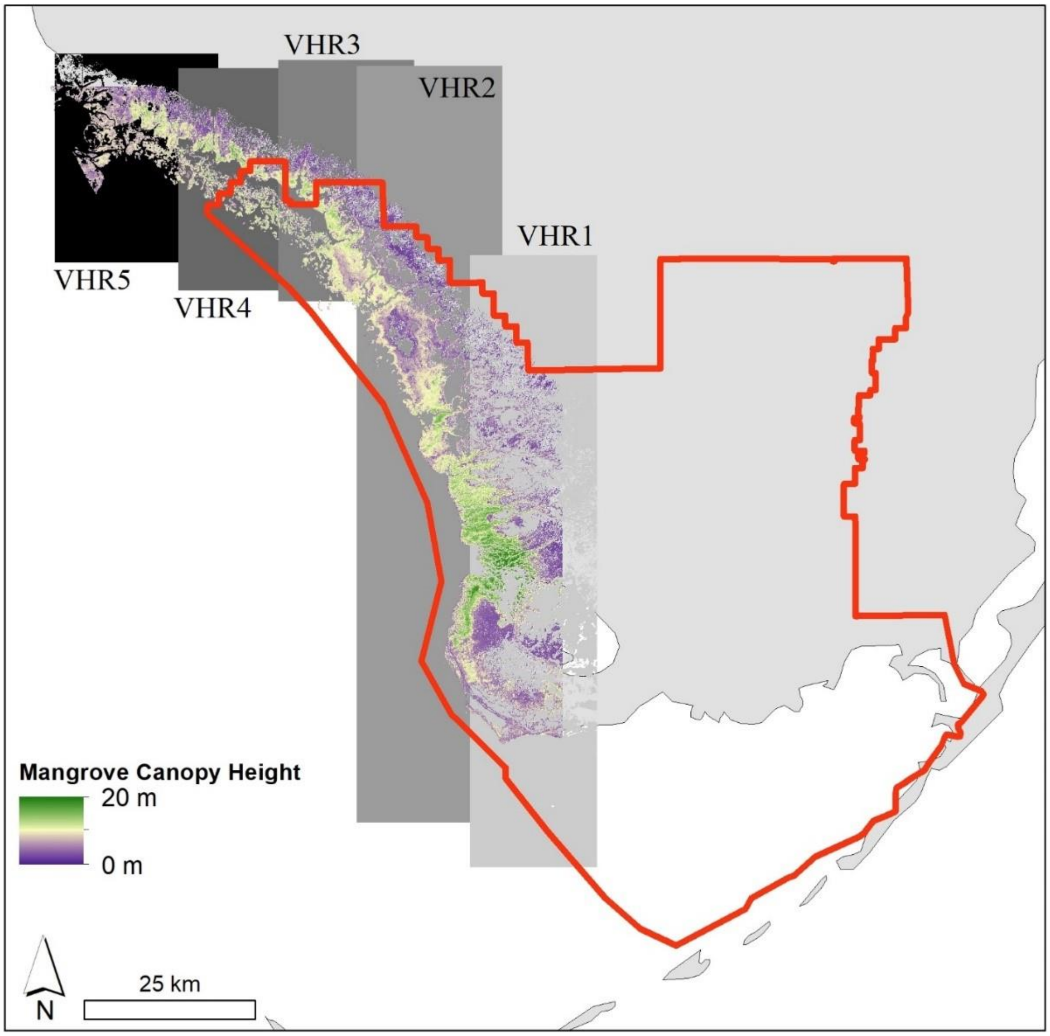

Supplementary Figure 6. Five Worldview Digital Surface Models were used to estimate mangrove canopy heights across the study domain in southwest Florida. The boundary of Everglades National Park is shown in red. Each labeled image corresponds to values in Supplementary Figure 5.

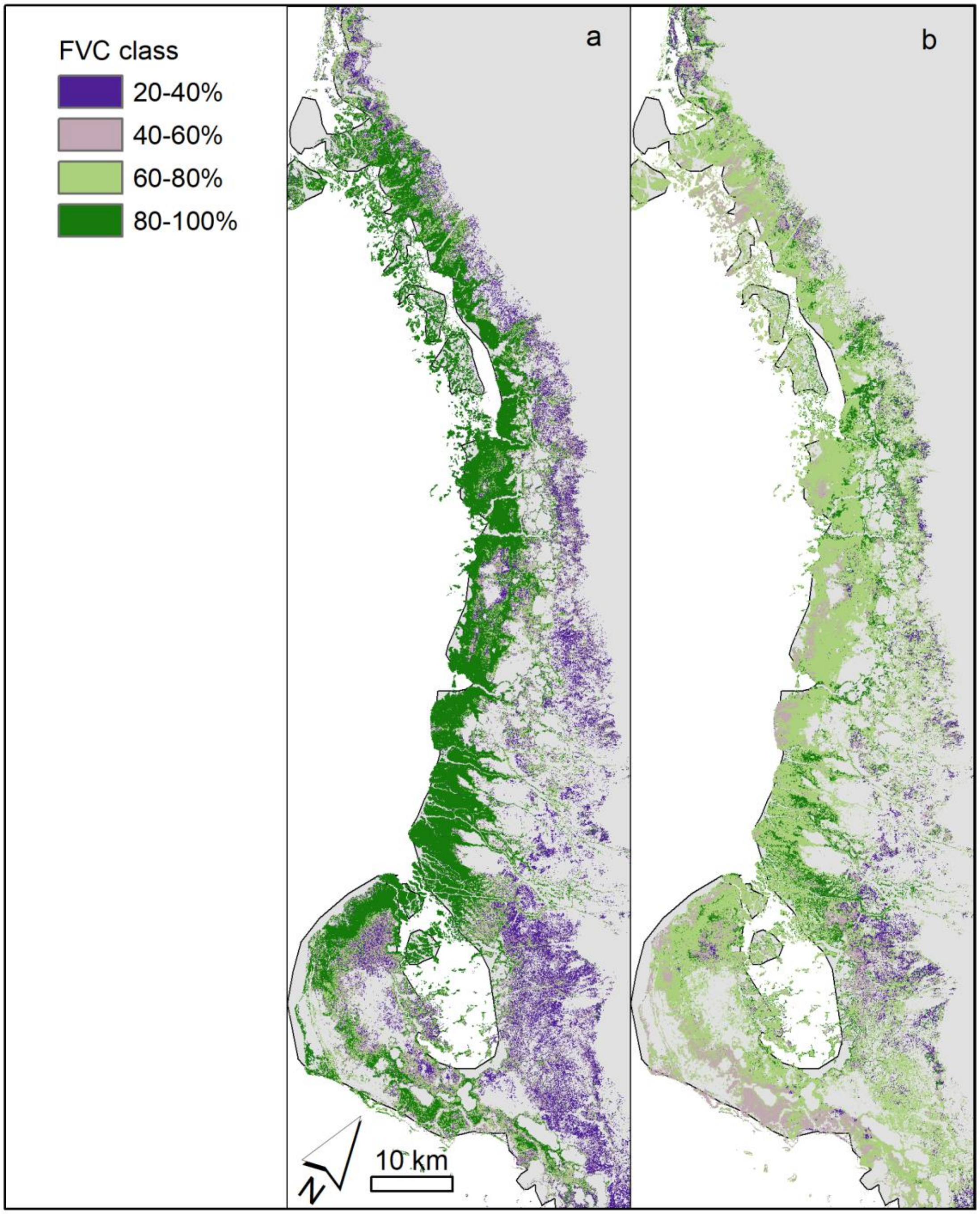

Supplementary Figure 7. For each time period, FVC was calculated from Landsat imagery calibrated using G-LiHT airborne lidar data. Mangrove fractional vegetation cover (FVC) before (a) and after (b) Hurricane Irma.

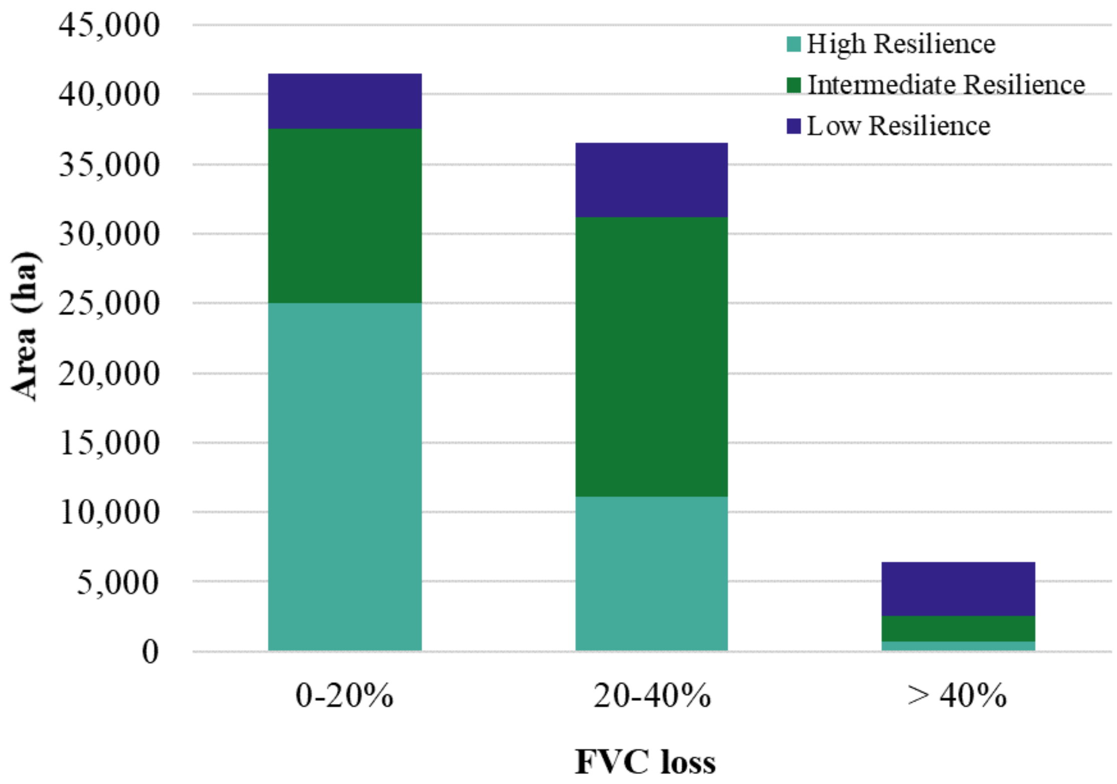

Supplementary Figure 8. Mangrove fractional vegetation cover (FVC) loss within each recovery class type. Areas where FVC loss exceeded 40%, had a higher chance of resulting in low resilience. However, low resilience was measured regardless of FVC loss.

Supplementary Tables

Ecosystem Structure	Resilience Class	Kolmogorov-Smirnov KS	1st quartile	3rd quartile	KScrit
Pre-storm canopy height WorldView-2 Stereo	Low-Intermediate	0.242	0.228	0.256	0.061
	Low-High	0.228	0.214	0.242	0.061
	Intermediate-High	0.294	0.280	0.306	0.061
Surface elevation National Elevation Data (NED)	Low-Intermediate	0.210	0.200	0.218	0.061
	Low-High	0.398	0.386	0.412	0.061
	Intermediate-High	0.230	0.218	0.240	0.061
Storm surge above ground Coastal Emergency Risks Assessment and National Elevation Data (CERA-NED)	Low-Intermediate	0.192	0.180	0.206	0.061
	Low-High	0.346	0.332	0.362	0.061
	Intermediate-High	0.376	0.364	0.390	0.061
CHM loss - total NASA G-LiHT lidar	Low-Intermediate	0.094	0.080	0.108	0.061
	Low-High	0.302	0.288	0.316	0.061
	Intermediate-High	0.342	0.328	0.356	0.061
CHM loss - percent NASA G-LiHT lidar	Low-Intermediate	0.058	0.048	0.068	0.061
	Low-High	0.286	0.274	0.300	0.061
	Intermediate-High	0.258	0.244	0.272	0.061

Supplementary Table 1. Two-sided Kolmogorov-Smirov statistics (KS) and critical KS statistics (KScrit) were generated to determine whether distributions between classes were significant. The median and first and third quartiles KS were determined from 5000 runs each using 500 randomly sampled points.

a

		Maximum Wind Classes (kph)					
		25-30	30-35	35-40	40-45	45-50	>50
Canopy Height Classes (meters)	>20		0.418	0.305	0.048	0.103	
	15-20	0.394	0.137	0.076	0.021	0.015	
	10-15	0.116	0.049	0.033	0.019	0.009	0.074
	5-10	0.057	0.034	0.013	0.010	0.006	0.019
	0-5	0.030	0.015	0.003	0.004	0.003	0.014

b

		Maximum Wind Classes (kph)						
		25-30	30-35	35-40	40-45	45-50	>50	Total
Canopy Height Classes (meters)	>20	0.00	0.54	8.73	103.41	23.58	0.00	136.26
	15-20	4.14	32.31	88.74	530.28	1043.64	0.00	1699.11
	10-15	38.79	99.27	242.91	760.32	2470.32	3.60	3615.21
	5-10	67.77	123.84	604.80	1192.95	3057.30	122.94	5169.60
	0-5	62.19	120.33	2255.85	1477.89	3702.60	168.39	7787.25
	Total	172.89	376.29	3201.03	4064.85	10297.44	1294.93	18407.43

Supplementary Table 2. Standard errors of estimated height losses from Hurricane Irma were highest for where the areal coverage was less than 50 ha. (a) Canopy loss standard errors in meters. (b) Areal extent in hectares covered by NASA G-LiHT lidar data where larger extent represents more observations. Prior to Hurricane Irma, the majority of mangrove forests in south Florida were ≤ 10 m. Therefore, while taller forests were more vulnerable to damage from hurricane-force winds, the majority of the region experienced intermediate impacts to canopy height and fractional vegetation cover.
