# Supplementary material for: Storm surge and ponding explain mangrove dieback in southwest Florida following Hurricane Irma
Source: Nat Commun. 2021 Jun 28;12:4003. doi: 10.1038/s41467-021-24253-y (PMC8238932; doi:10.1038/s41467-021-24253-y)
Supplement: Supplementary file 3 — Reporting Summary [file 41467_2021_24253_MOESM3_ESM.pdf]

nature research

Corresponding author(s): David Lagomasino

Last updated by author(s): May 17, 2021

Reporting Summary

Nature Research wishes to improve the reproducibility of the work that we publish. This form provides structure for consistency and transparency in reporting. For further information on Nature Research policies, see our Editorial Policies and the Editorial Policy Checklist.

Statistics

For all statistical analyses, confirm that the following items are present in the figure legend, table legend, main text, or Methods section.

n/a	Confirmed	
☐	☒	The exact sample size (*n*) for each experimental group/condition, given as a discrete number and unit of measurement
☐	☒	A statement on whether measurements were taken from distinct samples or whether the same sample was measured repeatedly
☐	☒	The statistical test(s) used AND whether they are one- or two-sided<br>*Only common tests should be described solely by name; describe more complex techniques in the Methods section.*
☐	☒	A description of all covariates tested
☐	☒	A description of any assumptions or corrections, such as tests of normality and adjustment for multiple comparisons
☐	☒	A full description of the statistical parameters including central tendency (e.g. means) or other basic estimates (e.g. regression coefficient) AND variation (e.g. standard deviation) or associated estimates of uncertainty (e.g. confidence intervals)
☐	☒	For null hypothesis testing, the test statistic (e.g. *F*, *t*, *r*) with confidence intervals, effect sizes, degrees of freedom and *P* value noted<br>*Give P values as exact values whenever suitable.*
☒	☐	For Bayesian analysis, information on the choice of priors and Markov chain Monte Carlo settings
☒	☐	For hierarchical and complex designs, identification of the appropriate level for tests and full reporting of outcomes
☐	☒	Estimates of effect sizes (e.g. Cohen's *d*, Pearson's *r*), indicating how they were calculated

Our web collection on statistics for biologists contains articles on many of the points above.

Software and code

Policy information about availability of computer code

Data collection	We used Google Earth Engine to acquire Landsat 7 and 8 Tier-1 Surface Reflectance imagery. Riegl's software, RiACQUIRE version 2.3.7, provides a graphical user interface for scanner control and near real-time monitoring of scanning LiDAR and GPS-INS data during lidar data collection.
Data analysis	This study used a combination of open-source code available in Google Earth Engine and R (version 4.0.3). Spatial Analyst Tools within ArcMap (version 10.7.1) were also used. The Ames Stereo Pipeline version 2.5.1 was used to process the WorldView-2 stereo satellite imagery.

For manuscripts utilizing custom algorithms or software that are central to the research but not yet described in published literature, software must be made available to editors and reviewers. We strongly encourage code deposition in a community repository (e.g. GitHub). See the Nature Research guidelines for submitting code & software for further information.

Data

Policy information about availability of data

All manuscripts must include a data availability statement. This statement should provide the following information, where applicable:

- Accession codes, unique identifiers, or web links for publicly available datasets
- A list of figures that have associated raw data
- A description of any restrictions on data availability

All original datasets used in this study are freely or commercially available through their respective references and data portals, with the exception of the wind data. Original final data products generated and used in this study are archived at PANGAEA Data Publisher, https://doi.pangaea.de/10.1594/PANGAEA.920522. NASA G-LiHT canopy height and fractional vegetation cover data is available through the NASA G-LiHT web portal, https://glihtdata.gsfc.nasa.gov/. The Hurricane Irma maximum storm surge models are available at https://cera.coastalrisk.live/. The National Elevation Datasets are available at https://apps.nationalmap.gov/viewer/. NASA GMAO maximum wind data used is available upon reasonable request.

Field-specific reporting

Please select the one below that is the best fit for your research. If you are not sure, read the appropriate sections before making your selection.

☐ Life sciences ☐ Behavioural & social sciences ☒ Ecological, evolutionary & environmental sciences

For a reference copy of the document with all sections, see nature.com/documents/nr-reporting-summary-flat.pdf

Ecological, evolutionary & environmental sciences study design

All studies must disclose on these points even when the disclosure is negative.

Study description	Mangroves buffer inland ecosystems from hurricane winds and storm surge. However, their ability to withstand harsh cyclone conditions depends on plant resilience traits and geomorphology. Using airborne lidar and satellite imagery collected before and after Hurricane Irma, we estimated that 62% of mangroves in southwest Florida suffered canopy damage, with largest impacts in tall forests (>10 m). Mangroves on well-drained sites (83%) resprouted new leaves within one year after the storm. Whereas, in poorly drained inland sites, Irma triggered one of the largest mangrove diebacks ever recorded (10,760 ha). The combination of low elevation (median = 9.4 cm asl), storm surge water levels (>1.4 m above the ground surface), and hydrologic isolation disproportionately impacted forests dominated or co-dominated by Avicennia germinans (73%). Our results show that storm surge and ponding caused dieback, not wind.
Research sample	A combination of airborne and satellite remote sensing data were used to quantify changes in mangrove forest structure and function from Hurricane Irma. Findings based on multi-sensor airborne data were scaled to the entire study area using estimates of forest structure and vegetation phenology derived from satellite data. We collected airborne lidar data before (April 2017) and after (December 2017) the storm with NASA Goddard's Lidar, Hyperspectral, and Thermal (G-LiHT) airborne imager to estimate the 3D changes in vegetation structure at 1-m spatial resolution across 130,000 ha of coastal wetlands in south Florida. We combined the GLiHT data with high-resolution satellite stereo imagery and Landsat time series information to track the recovery of mangrove forests across gradients of canopy structure damage, exposure to maximum hurricane winds, storm surge, community composition, and ground elevation. By intersecting the measured structural damage and recovery trajectories with species composition maps and topographic elevation models we were then able to identify the role of wind and storm surge on mangrove forests, including evidence for the main drivers of long-term dieback.
Sampling strategy	The sampling strategy to collect airborne lidar data was based on several factors that included aerial distribution of mangrove forests across southwest Florida, long-term ground monitoring stations, and a gradient of canopy height and species composition types. Within the area of airborne lidar data we selected all available satellite stereo imagery from WorldView-2. Lidar data was collected over 130,000 ha of coastal wetlands, with the majority in mangrove forests, during the flight campaign in April 2017. These same flight lines were sampled again in December 2017. This collection of lidar information is one of the largest databases for mangrove forests, particularly within 8 months and following a catastrophic cyclone.
Data collection	G-LiHT data were collected by co-authors Bruce Cook and Lawrence Corp. Airborne data collection followed standard NASA protocols for collecting lidar data over forests. The VQ-280 (Riegl USA, Orlando, FL, USA) airborne laser scanner mounted on a small aircraft was used to collect lidar data over mangrove forests. Lidar data was acquired using the RiACQUIRE version 2.3.7 software. The plane flew at a nominal height of 335 m above aground level at a pulse repetition frequency of 300 kHz to collect ~12 laser pulses per square meter. Flight lines targeted strategic areas of the mangrove forests in south Florida covering a range of canopy heights and coastal environments. All Landsat imagery was acquired through Google Earth Engine and cloud/shadow masks were applied to remove invalid pixels. WorldView 2 satellite stereo imagery was acquired through the NASA NextView License agreement with Maxar Inc.
Timing and spatial scale	G-LiHT lidar data were collected in April and December of 2017. Data collected in April coincided with the south Florida dry season. Data collected in December was collected as soon as possible, a combination of funding and mobilization, in order to capture the near-immediate effects of the storm. A baseline Landsat composite (pre-storm) was generated from August 31, 2015 through August 31, 2017. This baseline period was used in order to include the variability in spectral reflectance over a two-year period with no significant storm impacts. Fractional Vegetation Cover loss was estimated between the baseline Landsat image and a post-storm Landsat composite image from October 1, 2017 and December 31, 2017. The period coincided with the time period after the hurricane made landfall and the collection of the G-LiHT lidar data. Recovery estimates were determined using Landsat observations between January 2018 and December 2018, in order to estimate the recovery of forests in the year after a catastrophic hurricane.
Data exclusions	No data was excluded.
Reproducibility	All original datasets used in this study are all openly available, and all code used to generate the results come from open-sourced repositories. There were no replicated experiments. All available lidar data, stereo satellite imagery, and Landsat imagery over the mangrove forests during the given time periods were used in the analyses.
Randomization	For testing the significance between environmental variables (i.e., pre-storm canopy height, canopy height loss, percent canopy height loss, surface elevation, and storm surge water level above ground) we employed a two-sided Kolmogorov-Smirnov test 48 implemented in R (version 4.0.3). First, we created a multi-band stacked image which included each of the variable layers. Within each resilience class (i.e., Low, Intermediate, and High) with randomly selected 10,000-20,000 points using Google Earth Engine to sample from the environmental variables image. From that sample set we then randomly selected 500 samples within each of the resilience classes. Each class combination (1) Low-Intermediate, (2) Low-High, and (3) Intermediate-High were compared using the Kolmogorov-Smirnov test. We repeated this procedure using 5000 iterations in order to provide a robust estimate of the Kolmogorow-Smirnov statistic, including the mean and first and third quartiles, which were then compared to the critical value.

Blinding	Blinding was not used in our study. Training points for the mangrove cover model were selected using user-interpretation then compared with published inventory maps.

Did the study involve field work? ☐ Yes ☒ No

Reporting for specific materials, systems and methods

We require information from authors about some types of materials, experimental systems and methods used in many studies. Here, indicate whether each material, system or method listed is relevant to your study. If you are not sure if a list item applies to your research, read the appropriate section before selecting a response.

Materials & experimental systems

n/a	Involved in the study
☒	☐ Antibodies
☒	☐ Eukaryotic cell lines
☒	☐ Palaeontology and archaeology
☒	☐ Animals and other organisms
☒	☐ Human research participants
☒	☐ Clinical data
☒	☐ Dual use research of concern

Methods

n/a	Involved in the study
☒	☐ ChIP-seq
☒	☐ Flow cytometry
☒	☐ MRI-based neuroimaging
